# Editorial: Metals and cognitive decline: Pathophysiology, treatment, and prevention

**DOI:** 10.3389/fneur.2023.1150966

**Published:** 2023-03-20

**Authors:** Emel Koseoglu, Gang Liu, Agostinho Almeida

**Affiliations:** ^1^Faculty of Medicine, Erciyes University, Kayseri, Türkiye; ^2^Department of Radiology and Imaging Sciences, The University of Utah, Salt Lake City, UT, United States; ^3^Faculty of Pharmacy, University of Porto, Porto, Portugal

**Keywords:** metals, cognitive decline, air pollution, metal protein attenuating compounds, iron accumulation, overlapping pathologies of aberrant proteins, synaptic zinc, interaction between body and environment

This Research Topic aimed to capture new knowledge to understand the role of metals in cognition and cognitive disorders and to find effective measures for the treatment or prevention of cognitive decline in relation to metals.

In this Research Topic, an interesting collection of papers was presented, comprising the ones investigating molecular pathogenetic mechanisms related to metals and hypothalamic-pituitary-gonadal hormones in the modulation of metal ion homeostasis in cognitive dysfunction. The effect of metal-containing air pollutants on cognition in a young population was also examined. Excess iron in the brain has long been considered an important contributor to neurodegenerative diseases; however, the mechanisms underlying iron accumulation and the consequent action remain largely unknown. A review article provided some insights as well as new strategies for the treatment of neurodegenerative diseases. Additionally, the place of metal chelators in treatment and future directions in this area, with particular attention to a promising group of chelators, was presented in an opinion article. The importance of preventing air pollution was emphasized in the other opinion article.

The seven articles that make up this Research Topic are summarized below, in the sequence of their publication dates:

1. In the original research paper by Calderón-Garcidueñas et al., the cognitive states of 336 clinically healthy, middle-class Mexican volunteers, aged 29.2 ± 13 years, from three Mexican cities were examined and the assessments were performed with respect to the air pollution in living environments. Montreal Cognitive Assessment (MoCA) scores were found to be statistically lower in residents of Metropolitan Mexico City, which has high atmospheric levels of metal-rich fine particulate matter (PM_2.5_) and nanoparticles, compared to the residents of low-polluted cities. The authors strongly emphasize that exposures to air pollutants, particularly PM_2.5_ and their metal content, negatively impact cognitive abilities even in a young population.
2. In the opinion paper by Cukierman and Rey, the place of metal protein attenuating compounds, especially N-acylhydrazones in the potential treatment and/or prevention of metal-enhanced neuroaggregopathies, was discussed. The authors, being pioneers in studies related to N-acylhydrazones, compared the physicochemical and pharmacologic properties of these molecules in a timeline frame with other compounds and their effectiveness. In conclusion, they pointed out that these molecules are quite promising, and due to their versatility in terms of both affinity and specificity, by changing their ligand substituents, there is an important chance of improving new derivatives which makes them even more effective.
3. In the review by Rosenblum and Kosman, iron metabolism and its relationships to chronic inflammation and neurodegenerative diseases were presented along with related therapeutic interventions. The authors discussed two molecular mechanisms, explaining how brain iron accumulation can occur in inflammatory states based on functional changes in iron uptake and efflux proteins and changes in permeability. Appropriate combination therapy of antioxidants and iron chelators, such as N-acetyl cysteine and deferiprone, appears to be promising. More interestingly, some drugs such as mini hepcidins that can regulate iron transport across the blood-brain barrier are being developed.
4. In the original research by Liu et al., the aim was to assess the effects of age-related changes in hypothalamic-pituitary-gonadal hormones in the modulation of brain metal ion homeostasis. The authors measured the concentrations of brain metals in different female mice groups after ovariectomy and/or treatment with leuprolide or with no manipulation. Although there was a tendency for a decrease in all brain metals following ovariectomy, brain Cu was the only metal significantly correlated with plasma levels of LH and FSH. The brain concentrations of Zn and Cu, and Fe and Zn were positively correlated. The authors suggest that homeostatic mechanisms may have compensated for changes during chronic experimental manipulations. Short-term studies are needed.
5. In the opinion paper by Calderón-Garcidueñas, the neurological impact of air pollution was discussed. Giving examples from forensic autopsies of residents in a highly air-polluted city, the author pointed out that highly reactive solid atmospheric nanoparticles starting from a very early stage of life can cause aberrant neuronal aggregates and extensive organelle abnormalities in neural cells and progressive neurovascular damage since these particles can cross the placenta. The author suggests that the diagnostic criteria of neurodegenerative diseases should be overviewed since overlapping pathologies of aberrant proteins and other neuropathological changes are evident even in intrauterine life. Prevention, with environmental precautions, is the most important action to be taken immediately.
6. In the original research by Wang et al. involving a total of 9,527 subjects from the Chinese Hypertension Registry Study, the association between total plasma homocysteine (tHcy) levels and cognitive function among hypertensive patients was evaluated. Plasma tHcy levels had a threshold effect on Mini-Mental State Examination scores among hypertensive patients. Increased plasma tHcy levels were independently and inversely associated with cognitive decline among hypertensive patients with tHcy concentrations < 27.1 μmol/L.
7. In the original research by Vogler et al., the authors investigated the effect of disrupting Zn^2+^ modulation of neurotransmission in a transgenic mouse model lacking synaptic Zn^2+^ (ZnT3KO). They found age-dependent cognitive impairment, epileptiform activity on electroencephalogram, age-dependent increase in mossy fibers, and a decrease in synaptic density in hippocampal areas of the mice. Additionally, genetic removal of Zn^2+^ reduced the activity-dependent increase in Erk phosphorylation and brain-derived neurotrophic factor mRNA, and caused Erk activation by selective regulation of NMDA receptor subunits. The author discusses the importance of these changes with regard to amyloid pathology in Alzheimer's disease and their effects.

In summary, this Research Topic addressed, from various perspectives, the involvement of metals in the pathogenesis of cognitive dysfunction and the possibilities of treatment and prevention. As proven, in addition to their direct involvement in cognitive processes, metals play collaborative roles with the aberrant proteins in cognitive diseases. Moreover, as many metals are essential and some are toxic elements for the human body, they establish an interaction between the body and the environment, so the environment can easily affect brain health. We believe that metals deserve more attention to find effective treatments and preventive measures for cognitive disorders.

## Author contributions

EK: conceptualization, writing the original draft, and writing—review and editing. AA and GL: writing—review and editing. All authors contributed to the article and approved the submitted version.

