# Corrigendum: Editorial: Metals and cognitive decline: pathophysiology, treatment, and prevention

**DOI:** 10.3389/fneur.2023.1295167

**Published:** 2023-10-09

**Authors:** Emel Koseoglu, Gang Liu, Agostinho Almeida

**Affiliations:** ^1^Faculty of Medicine, Erciyes University, Kayseri, Türkiye; ^2^Department of Radiology and Imaging Sciences, The University of Utah, Salt Lake City, UT, United States; ^3^Faculty of Pharmacy, University of Porto, Porto, Portugal

**Keywords:** metals, cognitive decline, air pollution, metal protein attenuating compounds, iron accumulation, overlapping pathologies of aberrant proteins, synaptic zinc, interaction between body and environment

In the published article, there was an error in affiliation 2. Affiliation 2 “Department of Neurology, St Thomas' Hospital, London, United Kingdom” has been removed.

The authors apologize for this error and state that this does not change the scientific conclusions of the article in any way. The original article has been updated.

